# Hot-Air Furnaces

**Published:** 1882-02

**Authors:** 


### HOT-AIR FURNACES

OUGHT not to be tolerated; they ruin the wood-work of any building, ruin the furniture, and, more than all, impair the health of every person who breathes the atmosphere of houses thus heated by them. Warm air relaxes, debilitates, the world over; cool air braces up, gives tone, vigor, power, to the whole frame.

Warm air evaporates every article that has moisture in it, fluids, meats, vegetables—everything; these particles are distributed all through the air of the house, to the exclusion, to that extent, of the life-giving oxygen; so that not one single breath of pure air is taken into the lungs, as long as the person occupies such a house; and when it is remembered that during the most inclement season of the year, there are days, even weeks, during which the very young and the very old of the family, as also the invalids, do not pass outside the door, it is not to be wondered at that there is not one day, during all the winter, in which health dwells in any household so warmed. But a great deal of the ill effect of furnace-heated rooms may be obviated, if the fire-place is always kept open; but in very cold weather, there should be a fire in the fire-place, in order to create a more decided draught towards it, so as to promote a circulation, and carry the bad air more rapidly up through the chimney, and out of the building.

It is a great mistake, and an almost universal one, that sudden changes from one temperature to another are prejudicial to health. If persons will close their mouth, and send all the air to the lungs through the circuit of the head, and thus temper it to the air of the lungs, a positive benefit will result, although there may be a change of forty degrees in a second of time; only one precaution is needed. *Shut your mouth, and keep moving.*

The proof of all this is, railroad conductors are healthy men, as a class, and yet their changes are fifty degrees, hundreds of times in a day.

In addition, it is known to all persons of observation, that the inhabitants of the equable and moderate climates are not long-lived. The "Italian skies," and the "South of France,"

so much boasted of, do not give length of days to those who enjoy their balmy atmosphere.

Our grandsires lived in cozy parlors, and fire-place heated dining-rooms, with passages and halls as cold as Greenland, and yet they boast a higher health than their degenerate sons and daughters. These are facts, and they ought to have a rational consideration. Down, we say, with every hot-air furnace in the land!

---

### WARTS.

If they give you no special inconvenience, let them alone. But if it is of essential importance to get rid of them, purchase half an ounce of *muriatic acid*, put it in a broad-bottomed vial, so that it will not easily turn over; take a stick as large as the end of a knitting-needle, dip it into the acid, and touch the top of the wart with whatever of the acid adheres to the stick; then, with the end of the stick, rub the acid into the top of the wart, without allowing the acid to touch the well skin. Do this night and morning, and a safe, painless, and effectual cure is the result.

---

### HUNGER.

If a man in good health has not eaten anything for some days, he will die if he eats heartily. When persons are found in an almost starving condition, light food, in small quantities, and at short intervals, is essential to safety. The reason is, that as soon as we begin to feel hungry, the stomach rolls and works about, and continues to do so, unless satisfied, until it is so exhausted that there is scarcely any vital energy; it is literally *almost tired to death*, and, therefore, digestion is performed slowly, and with great difficulty. Hence, when a person has been kept from eating several hours beyond his usual time, instead of eating fast and heartily, he should take his food with deliberation, and only half as much as if he had eaten at the regular time. Sudden and severe illness has often resulted from the want of this precaution, and sometimes death has followed.

